# ^18^F-FET PET radiomics-based survival prediction in glioblastoma patients receiving radio(chemo)therapy

**DOI:** 10.1186/s13014-022-02164-6

**Published:** 2022-12-02

**Authors:** Tun Wiltgen, Daniel F. Fleischmann, Lena Kaiser, Adrien Holzgreve, Stefanie Corradini, Guillaume Landry, Michael Ingrisch, Ilinca Popp, Anca L. Grosu, Marcus Unterrainer, Peter Bartenstein, Katia Parodi, Claus Belka, Nathalie Albert, Maximilian Niyazi, Marco Riboldi

**Affiliations:** 1 Department of Medical Physics, LMU Munich, Garching, Germany; 2 Department of Radiation Oncology, University Hospital, LMU Munich, Munich, Germany; 3 Department of Neurology, School of Medicine, Technical University of Munich, Munich, Germany; 4 German Cancer Consortium (DKTK), Partner Site, Munich, Germany; 5 German Cancer Research Center (DKFZ), Heidelberg, Germany; 6 Department of Nuclear Medicine, University Hospital, LMU Munich, Munich, Germany; 7 Department of Radiology, University Hospital, LMU Munich, Munich, Germany; 8 Department of Radiation Oncology, Medical Center, Faculty of Medicine, University of Freiburg, Freiburg, Germany

**Keywords:** Quantitative image analysis, Radiomics, Survival analysis, Glioblastoma, Radiotherapy

## Abstract

**Background:**

Quantitative image analysis based on radiomic feature extraction is an emerging field for survival prediction in oncological patients. ^18^F-Fluorethyltyrosine positron emission tomography (^18^F-FET PET) provides important diagnostic and grading information for brain tumors, but data on its use in survival prediction is scarce. In this study, we aim at investigating survival prediction based on multiple radiomic features in glioblastoma patients undergoing radio(chemo)therapy.

**Methods:**

A dataset of 37 patients with glioblastoma (WHO grade 4) receiving radio(chemo)therapy was analyzed. Radiomic features were extracted from pre-treatment ^18^F-FET PET images, following intensity rebinning with a fixed bin width. Principal component analysis (PCA) was applied for variable selection, aiming at the identification of the most relevant features in survival prediction. Random forest classification and prediction algorithms were optimized on an initial set of 25 patients. Testing of the implemented algorithms was carried out in different scenarios, which included additional 12 patients whose images were acquired with a different scanner to check the reproducibility in prediction results.

**Results:**

First order intensity variations and shape features were predominant in the selection of most important radiomic signatures for survival prediction in the available dataset. The major axis length of the ^18^F-FET-PET volume at tumor to background ratio (TBR) 1.4 and 1.6 correlated significantly with reduced probability of survival. Additional radiomic features were identified as potential survival predictors in the PTV region, showing 76% accuracy in independent testing for both classification and regression.

**Conclusions:**

^18^F-FET PET prior to radiation provides relevant information for survival prediction in glioblastoma patients. Based on our preliminary analysis, radiomic features in the PTV can be considered a robust dataset for survival prediction.

## Background

Glioblastoma is the most aggressive primary brain tumor with a median survival of less than two years [1–4]. Combining surgery, chemotherapy and radiotherapy is the current standard of care, but prognosis remains poor because of the heterogeneous and complex pathogenesis [2]. Therefore, survival prediction based on images acquired before treatment may represent an important step towards a more personalized approach. In recent years, a lot of research has been done in this area with a strong focus on quantitative medical image analysis [5].

There is an increasing interest towards the use of ^18^F-Fluorethyltyrosine (^18^F-FET) Positron Emission Tomography (PET) imaging for the characterization of aggressive brain tumors such as glioblastoma. ^18^F-FET PET images were reported to provide valuable information, able to effectively support treatment response assessment in glioblastoma [6–10]. In the framework of the recent randomized multicenter phase II trial ARTE, higher ^18^F-FET PET TBR (tumor-to-background ratio) on pre-treatment scans and persistent ^18^F-FET PET signal of longer contrast-enhancing tumor was found to associate with inferior overall survival [10]. However, dedicated studies are needed to assess the predictive power related to ^18^F-FET PET radiomics for survival prediction in glioblastoma. The effective use of radiomics to predict overall survival as an indicator of treatment response comes with a large variability of different strategies regarding image acquisition, tumor segmentation, image processing and feature extraction, data handling and implementation of machine learning models [5, 11, 12]. For example, reliable tumor segmentation methods should reduce the inherent intra- and inter-observer variability [13]. At the same time, segmented contours should be readily available to facilitate their integration in the treatment planning process. In addition, site-specific image acquisition protocols introduce variations in imaging scanners, image reconstruction and even SUV intensities, which can have an effect on the extracted radiomic features [12, 14, 15]. This calls for specific studies to evaluate the reproducibility of radiomic features, in order to maximize their use in survival prediction models.

The objective of this work was to identify key radiomic features in ^18^F-FET PET images acquired before treatment for glioblastoma patients undergoing radiochemotherapy, and to assess their reproducibility, aiming at consistent prediction accuracy of overall survival.

## Methods

Radiomic features were extracted from four different volumes of interest (VOIs) to identify the VOIs with the most reliable information in terms of survival prediction. After features were extracted, principal component analysis (PCA) was employed to reduce dimensionality of the feature space, working either as data reduction or feature selection method. Four different datasets were generated with the PCA and used as input data for the machine learning algorithms. Overall, three different prediction models were implemented: (1) a univariate Cox regression model to predict survival time, providing preliminary investigation of potentially relevant features, (2) a random forest classification model to predict overall survival at 1 year, (3) a random forest regression model to predict survival time.

### Clinical dataset

The retrospective study included 37 patients with glioblastoma (WHO grade 4) diagnosed between 2009 and 2017. Survival time was defined, in days, as the time between the day of treatment planning CT acquisition and the day of decease. Due to the high malignancy of glioblastoma and the related poor survival prognosis, the event of decease occurred in all of the cases. The following inclusion criteria were applied for patient selection:

- Evidence of macroscopic tumor before the start of treatment planning
- PET image acquired not more than 3 weeks before the treatment planning CT to avoid significant anatomical changes and tumor growth/shrinkage between PET imaging and start of treatment
- IDH-wildtype glioblastoma, in cases of available IDH mutational status
- Follow-up of at least one year.

^18^F-FET PET images were acquired at the Department of Nuclear Medicine, University Hospital of Munich prior to primary radiation treatment. ^18^F-FET PET images were used to delineate the tumor based on standardized uptake values (SUVs) and subsequently extract radiomic features within the selected volumes of interest (VOIs). Planning target volumes (PTVs) were delineated within the treatment planning software Oncentra External Beam (version 4.5, Nucletron, Elekta AB, Sweden). When available (32 out of 37 patients), the reconstructed treatment planning CTs and structures were restored from the archive using the treatment planning software.

^18^F-FET PET images acquired with a Siemens ECAT Exact HR+ (Siemens Healthineers, Erlangen, Germany) scanner according to the standard protocol of the department, described in detail previously [16], were used as initial training set (N = 25 cases). A transmission scan was performed during 15 min with a ^68^Ge rod source. Thereafter, approximately 180 MBq of ^18^F-FET were injected as an intravenous bolus. Once the patient was in position, a 40 min dynamic emission recording in 3D mode consisting of 16 frames was started. The ordered subset expectation maximization (OSEM) algorithm was used for image reconstruction slice per slice (OSEM-2D). The OSEM-2D reconstruction consisted of 6 iterations and used 16 projection subsets. The voxel size in images reconstructed with the described OSEM-2D algorithm was 2.035 × 2.035 × 2.425 mm^3^, with a resulting image matrix of size 128 × 128 × 63.

The remaining 12 patients were acquired in list mode with a Biograph 64 PET/CT (Siemens Healthineers, Erlangen, Germany), and OSEM-3D was used to reconstruct images. OSEM-3D images were reconstructed with 4 iterations, 21 subsets, a 5 mm Gaussian post-reconstruction filter and standard corrections (attenuation, random and scattered coincidences, dead time decay). The reconstruction matrix was set to 336 × 336 × 109, with voxel size 1.018 × 1.018 × 2.027 mm^3^.

The key features of the available clinical dataset are summarized in Table 1.

**Table 1:** Features of the clinical dataset

*Patient characteristics*	
Histopathologic diagnosis	
Glioblastoma, WHO grade 4	N = 33
Anaplastic astrocytoma, IDH wildtype, TERT promotor mutation (Glioblastoma, WHO grade 4 equivalent according to the current WHO classification)	N = 4
IDH mutational status	
IDH WT	N = 28
IDH mutational status unknown	N = 9
MGMT promotor methylation status	
Methylated	N = 21
Unmethylated	N = 16
Sex	
Male	N = 20
Female	N = 17
Age at the time of irradiation	
Median	62 years
Range	30–76 years
Neurosurgical treatment prior to irradiation	
Stereotactical biopsy only	N = 27
Neurosurgical resection	N = 10
Radiotherapy dosage	
30 × 2 Gy	N = 32
29 × 2 Gy (due to discontinuation)	N = 1
33 × 1.8 Gy	N = 2
12 × 1.8 Gy/2,1 SIB (due to discontinuation)	N = 1
10 × 3 Gy plus 1 × 4 Gy	N = 1
Chemotherapy concomitant to radiotherapy	
Temozolomide (EORTC26981/22981-NCIC CE3 protocol)	N = 34
Temozolomide and lomustine (NOA-09 protocol)	N = 1
No concomitant chemotherapy	N = 2
*PET characteristics*	
PET acquisition protocol	
Intravenous bolus	180 MBq
Scan duration	40 min
Dynamic frames	16
OSEM-2D	
Iterations	6
Subsets	16
Voxel spacing	2.035 × 2.035 × 2.425 mm^3^
Reconstruction matrix	128 × 128 × 63
OSEM-3D	
Iterations	4
Subsets	21
Voxel spacing	1.018 × 1.018 × 2.027 mm^3^
Reconstruction matrix	336 × 336 × 109
Post-reconstruction filter	Gaussian (5 mm)

### Feature extraction

Four different volumes of interest (VOIs) were used to compare the prognostic power of the radiomics analysis when limited to a specific region. The PMOD software (PMOD Technologies, Zurich, Switzerland) was used to generate PET-based VOIs and to register PTV contours to the PET images. PET-based VOIs were defined using a semi-automatic segmentation method, as described by Unterrainer et al. [16]. A tumor-to-background ratio (TBR) threshold of 1.6 has been considered to be optimal to differentiate tumor tissue and surrounding healthy tissue [16, 17]. Besides 1.6, two additional TBR thresholds were chosen, one smaller value of 1.4 and one larger value of 1.8. This procedure resulted in a set of three PET-based VOIs for every patient, further on referred to as *voi14*, *voi16* and *voi18* corresponding to the TBR threshold 1.4, 1.6 and 1.8, respectively.

**Fig. 1:**
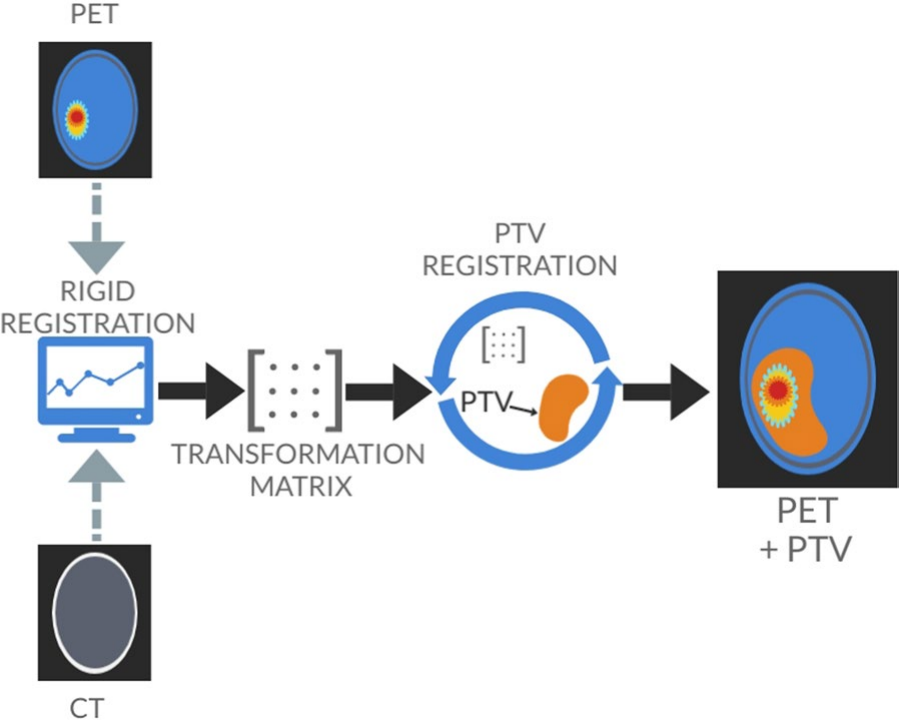
Workflow of the image registration procedure that was implemented to overlay the PTV and PET-based VOIs onto the FET-PET image

The fourth VOI was generated using the PTV, which was defined by experienced radiation oncologists during the treatment planning process. The gross tumor volume (GTV) was contoured based on the contrast enhanced T1 weighted MRI, complementing the treatment planning CT. T2 and Fluid Attenuated Inversion Recovery (FLAIR) MRI images were used in addition if available. The clinical target volume (CTV) was then created by adding a 2 cm margin to the GTV [18]. The final planning target volume (PTV) was obtained by adding 0.3–0.5 cm to the CTV. As the goal was to extract radiomics features from the PET image, the PTV structures had to be registered to the PET images. The PET image was loaded into PMOD, serving as reference image, whereas the treatment planning CT image was iteratively registered via rigid matching. The resulting transformation matrix was applied to the PTV contour to be overlaid onto the PET image. This provided PET intensity values within the PTV with no need to interpolate raw image intensities. The workflow of the image registration procedure is depicted in Fig. 1.

The PTV, when defined on the treatment planning CT, may include bone and other structures with high CT numbers that do not provide any information about tracer uptake in tumor tissue and surrounding brain tissue. As those regions would cause noise in the radiomic features and impair identification of intensity related patterns in tumor regions, these structures were excluded from the PTV. As shown schematically in Fig. 1, the PTV was much larger compared to the metabolically active tumor, consistently with previously reported studies [19]. This provided a way to assess the role of areas featuring a low intensity ^18^F-FET PET signal, included in the PTV but outside the metabolically active area (TBR-based VOIs).

Image intensities were rebinned using a fixed bin width approach [11, 20]. To determine the appropriate bin width, the mean TBR intensity range of the initial 25 patients was calculated from *voi14* and subsequently divided by the number of bins. Such a number was selected aiming at optimal feature robustness, as investigated by Tsujikawa et al. for PET imaging [21], and therefore set to 64. According to this strategy, a bin width of 0.04 was obtained for TBR-based resampling, by dividing the mean TBR intensity range (*voi14*) in 64 bins.

Following rebinning, the python package *pyradiomics* was used to extract quantitative image features for the selected VOIs (i.e. *voi14*, *voi16*, *voi18* and PTV) in the ^18^F-FET PET image [22, 23]. In total 107 radiomic features were extracted from the 3D PET images, including the following feature classes: First Order (FO), Shape (SH), Gray Level Co-occurrence Matrix (GLCM), Gray Level Size Zone Matrix (GLSZM), Gray Level Run Length Matrix (GLRLM), Neighbouring Gray Tone Difference Matrix (NGTDM), Gray Level Dependence Matrix (GLDM).

### Dimensionality reduction and feature selection

Unsupervised methods were applied to reduce the data dimensionality and perform feature selection. For both tasks we applied PCA based on the singular value decomposition (SVD) of the feature set, as provided within the scikit-learn package (version 0.22.2) [24–26]. PCA was utilized following the standardization of radiomic features, which resulted in feature values with zero mean and unit variance.

Four different types of feature datasets were considered:

- *Top*: the dataset consisted of 10 features per principal component and was generated using PCA-based feature selection: the features with the highest PCA loadings were retained, focusing on the components that explained 90–95% of the total variance in the data
- *PC*: the dataset consisted of the principal component values of each patient, calculated with the entire feature set of 107 features, aiming at the same total variance applied for the *Top* features
- *SH-FO*: the dataset consisted of all the shape and first order features (32 features overall)
- *PC(SH-FO)*: the dataset consisted of the principal component values of each patient, calculated with shape and first order features (32 overall), with the same goal in terms of total variance explained.

### Identification of relevant radiomic features

The extracted radiomic features were analyzed to highlight the relevant ones for the purpose of survival prediction.

The Cox proportional hazard model was applied to identify relevant radiomic features in survival prediction based on the calculated hazard ratios. The Cox regression was implemented using the *lifelines* package in python (version 0.24.4) [27]. Univariate Cox models were fitted to the four feature datasets and potentially relevant features were identified based on the reported p-value of the Wald statistics. A threshold corresponding to 95% significance (i.e. 5% p-value) was selected to classify whether the corresponding radiomic features had a statistically relevant impact on survival prediction.

### Survival prediction based on multiple features

We used random forests for both classification and regression analysis based on multiple radiomic features. Random forest is an ensemble method, which means that it combines a number of base estimators built with decision trees in order to reduce variance and improve generalizability [28, 29]. Random forest algorithms were implemented with the python package Scikit-learn [29]. Survival prediction was applied for classification (categorize survival at one-year follow-up) and regression (prediction of survival time). In Scikit-learn, the random forest algorithm for classification provided the use of different class weights to adjust for the imbalance in class frequencies.

To select the best hyper-parameter settings in the models, a coarse to fine strategy was applied using a randomized search cross-validation on training data. To reduce computation time, the randomized search cross-validation was applied twice: (1) in a very broad range of hyper-parameters to identify the order of magnitude of the best performing hyper-parameters and (2) once the magnitude was assessed, multiple values within this magnitude were included in the final parameter grid.

The performance in survival prediction was quantified on the optimal model (i.e. with the determined hyperparameters): we rated survival classification at 12 months based on the Area Under the Receiver Operating Characteristic Curve (AUC), and survival time prediction based on the Concordance Index (C-index). We considered different testing scenarios, where data from the 25 OSEM-2D cases were mixed with the 12 OSEM-3D cases:

1. Only OSEM-3D data in the test dataset, only OSEM-2D data in the training dataset
2. A fivefold train and test cross-validation using the entire dataset (OSEM-2D and OSEM-3D data combined).

## Results

### Patients and treatment

Patients were treated with conventionally fractionated radiochemotherapy with temozolomide (TMZ) based on the EORTC26981/22981-NCIC CE3 protocol [30] in 34 cases, in one case with conventionally fractionated radiochemotherapy with TMZ and lomustine according to the NOA-09 protocol [31], in one case with hypofractionated radiotherapy only and in one case with conventionally fractionated radiotherapy only due to a contraindication for chemotherapy. Radiotherapy dosage was 30 × 2 Gy in 32 cases, 29 × 2 Gy due to discontinuation in one case, 33 × 1.8 Gy in two cases, 12 × 1.8 Gy/2.1 Gy on a simultaneous integrated boost (SIB) due to discontinuation in one case and with 10 × 3 Gy plus 1 × 4 Gy in one case.

### Identification of relevant radiomic features

**Fig. 2:**
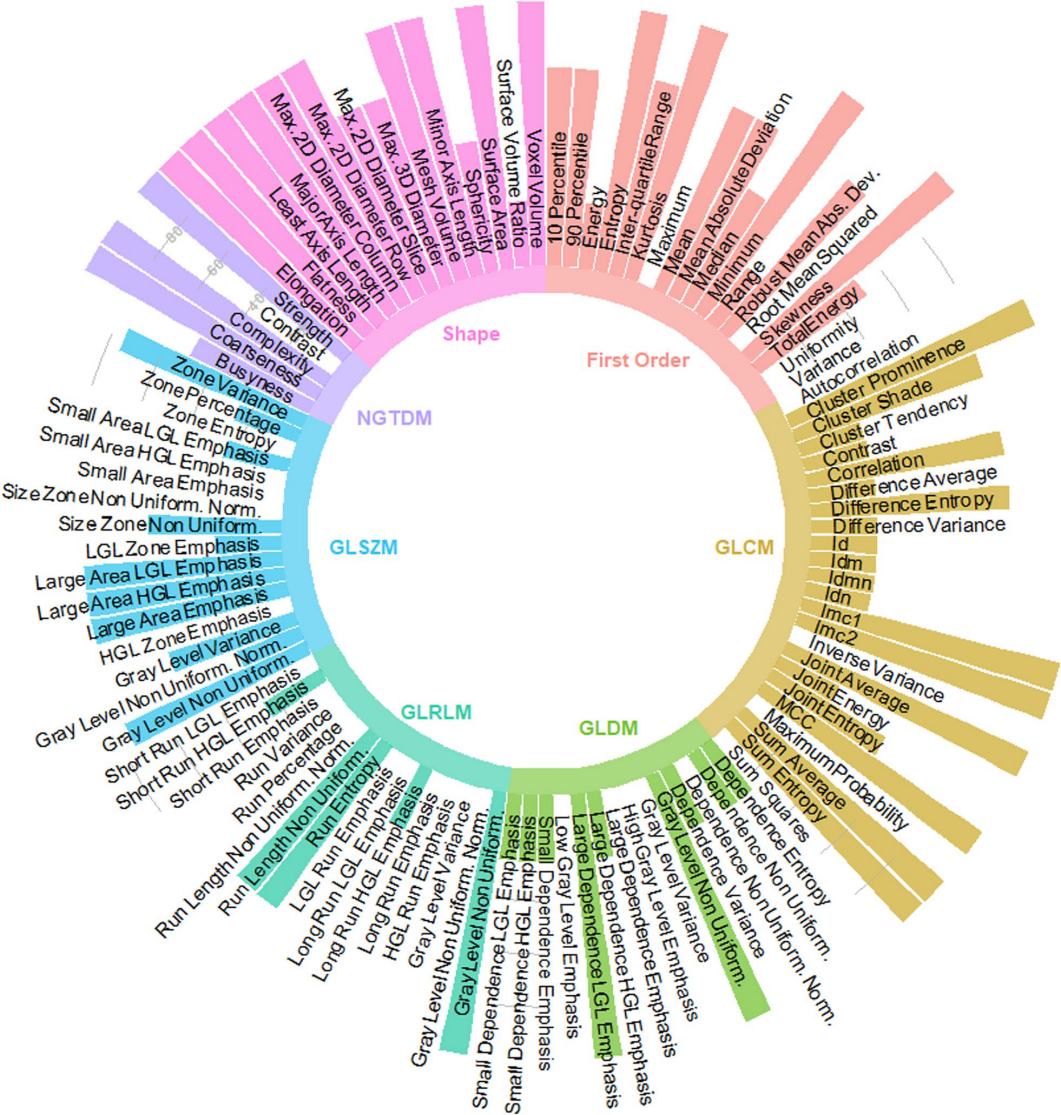
Circle plot showing the frequency of feature selection in the Top dataset. The height of each color bar in the circular plot is proportional to the selection frequency across the 4 VOIs. Color codes denote different radiomic features types, as indicated by text labels in the inner part of the circle

When considering the initial 25 patients, a total number of 5–7 principal components turned out to be sufficient to explain 90–95% of the total variance in the data. The *Top* dataset included the 10 features with the highest loadings for the first 5–7 principal components: Fig. 2 shows the features that appeared most frequently in the *Top* dataset across the 4 VOIs (*voi14*, *voi16*, *voi18* and PTV). Shape and first order features were predominantly selected (87.5% and 55.6% average selection frequency, respectively) compared to all other feature types (37.3% average selection frequency). This indicates that shape and first order features have larger variations across the analyzed glioblastoma patients, which might be relevant for outcome prediction.

**Fig. 3:**
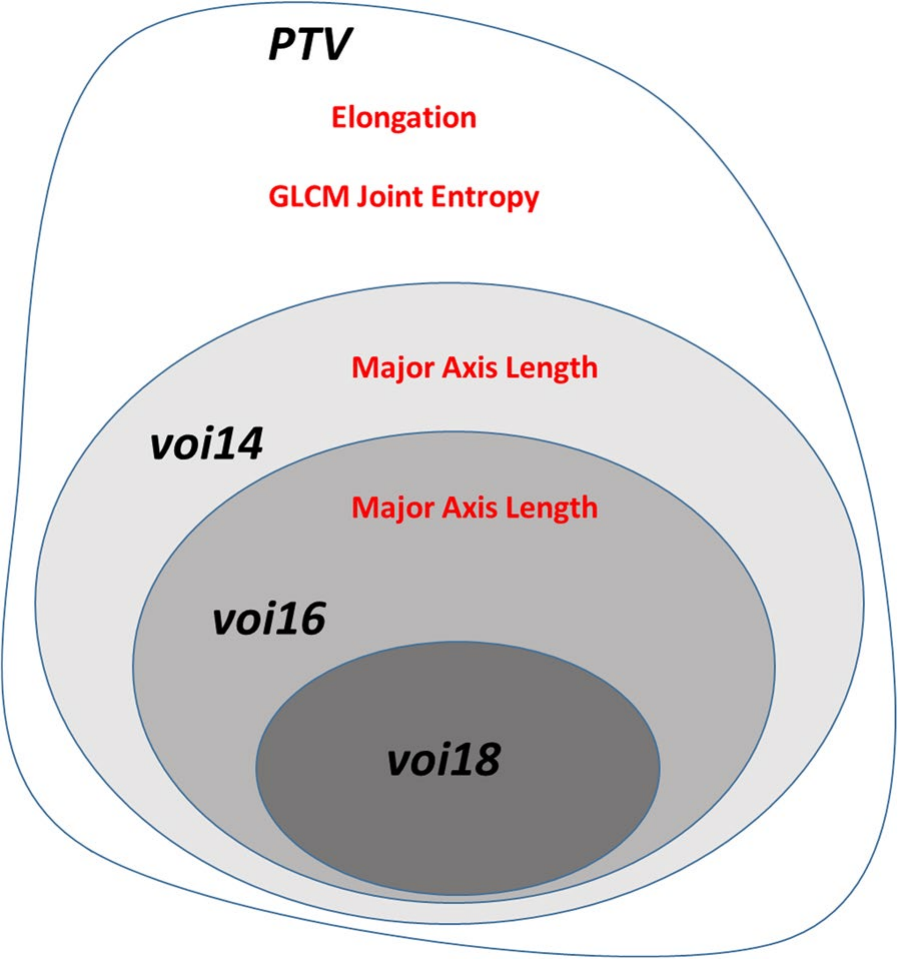
Statistically relevant features from the univariate Cox model fit for the *Top* radiomics dataset. All of the listed features were associated to a hazard ratio larger than 1, meaning that increasing values reduce the survival probability

When analyzing the statistical relevance of single radiomic features via the Cox proportional hazard model, very few radiomic features turned out to be significant in the *Top* dataset. Figure 3 depicts graphically which radiomic features were classified as significant in the different regions at 95% confidence in univariate Cox proportional hazard models.

**Fig. 4:**
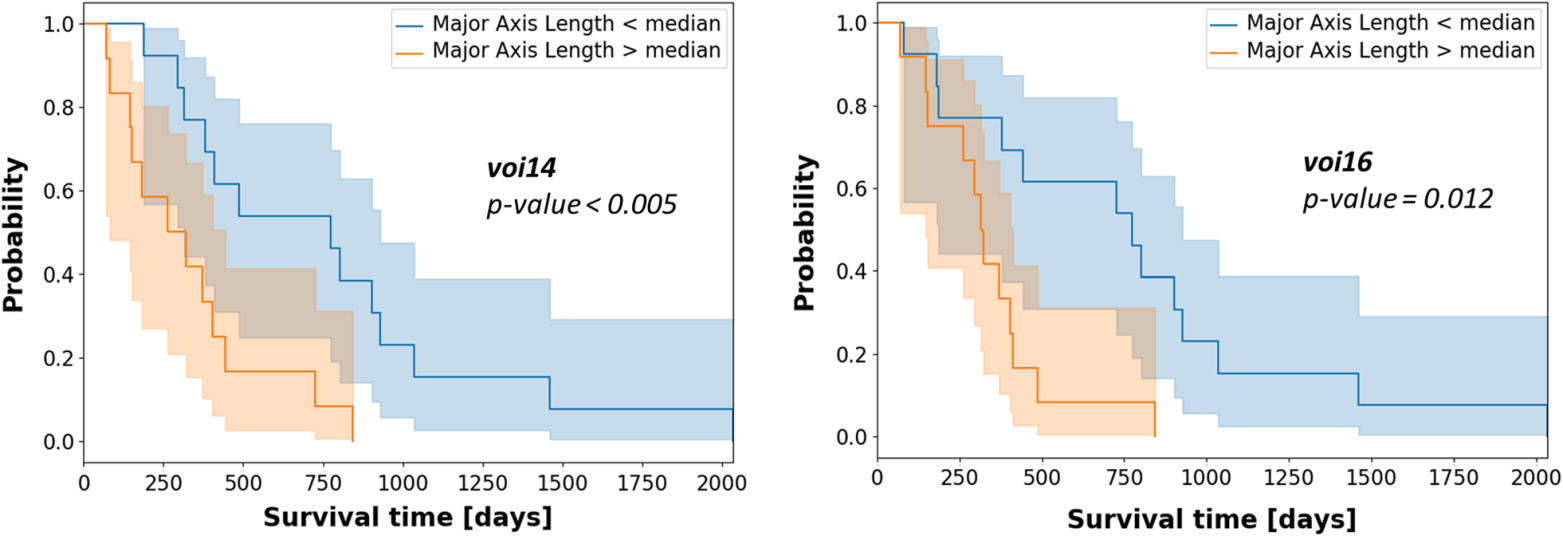
Kaplan–Meier survival curves showing the effect of the shape feature Major Axis Length in *voi14* (left panel) and *voi16* (right panel). Patient stratification based on the median value is depicted, with the corresponding p-value of the log-rank test

Figure 4 shows the effect on survival of the shape feature Major Axis Length in *voi14* and *voi16* which resulted in significantly different prediction performance for the univariate Cox proportional hazard model. The log-rank test comparison between the Kaplan–Meier curves, where patients were stratified according to the median value, exhibited a p-value smaller than 0.05. This indicates the ability of the radiomic feature Major Axis Length to stratify patients in different risk classes, connected to a significantly different probability of survival at 95% statistical confidence.

### Survival prediction based on multiple features

Table 2 reports the average results for the two testing scenarios. The best performance was obtained when considering the PTV as VOI for independent testing on OSEM-3D images. Conversely, results for the CV case were in general lower than independent testing. This indicates that features selected on OSEM-2D images can adequately predict survival in OSEM-3D, whereas data mixing in training does not allow the implemented models to achieve satisfactory prediction performance.

**Table 2:** Results on the testing experiments, expressed as average over all scenarios (1–4)

	CLASSIFICATION-AUC				REGRESSION-C-index			
	Top	PC	PC(SH-FO)	SH-FO	Top	PC	PC(SH-FO)	SH-FO
*Test on OSEM-3D*								
PTV	**0.76**	0.34	**0.77**	**0.80**	**0.76**	0.60	**0.68**	**0.67**
voi14	041	0.44	0.44	0.63	0.55	0.64	0.43	0.60
voi16	0.33	0.37	0.42	0.50	0.55	0.40	0.52	0.60
voi18	0.12	0.30	0.42	0.32	0.31	0.47	0.33	0.47
*CV OSEM-2D + OSEM-3D*								
PTV	0.59	0.29	**0.70**	0.58	0.51	0.47	0.62	0.53
voi14	0.56	0.51	0.44	0.59	0.59	0.52	0.50	0.53
voi16	0.26	0.32	0.39	0.50	0.55	0.51	0.45	0.65
voi18	0.57	0.48	0.40	0.39	0.43	0.44	0.19	0.42

Classification and regression performance figures above 65% are highlighted in bold

## Discussion

We analyzed the reproducibility of radiomic features extracted from ^18^F-FET PET images, and their use for survival analysis in glioblastoma patients. Two different patient cohorts were available which included cases with PET images reconstructed via an OSEM-2D algorithm, as well as more recent cases with OSEM-3D reconstruction. The OSEM-2D cohort served as a homogeneous training dataset, providing a consistent source of data to optimize the performance of survival prediction models based on multiple radiomic features. The random forests survival models, featuring the parameters optimized on the first patient cohort, were then tested in multiple scenarios including newer data. This provided a framework to check the reproducibility of radiomic features extracted from ^18^F-FET PET images to achieve accurate survival prediction in glioblastoma patients.

When analyzing the role of single radiomic features, shape and first order features extracted from ^18^F-FET PET images were highlighted as the most relevant in the analyzed dataset. This was confirmed both in unsupervised PCA feature selection (Fig. 2) and in the analysis of hazard ratios in univariate Cox proportional models for PET-based VOIs (Fig. 3). As visible in Fig. 3, different features were deemed significant when analyzing the univariate Cox proportional models in multiple regions (VOIs). Univariate Cox models were used to assess significant variables, instead of multivariate analysis. This choice was justified by the intrinsic limitations in Cox proportional models for reduced number of events per variable, as discussed in [32].

A closer look at these results highlights that increasing values of the shape feature Major Axis Length (i.e. the maximal extension of the biologically active area in the ^18^F-FET PET image) in *voi14* and *voi16* are significantly correlated to a reduced overall survival time. This means that larger regions of biologically active tumor in the ^18^F-FET PET image, when the image is thresholded at 1.4 or 1.6 TBR, are clear indicators of lower survival probability. When extending the analysis to the PTV, the Elongation was also found to be significantly correlated to survival. The Elongation is defined as the square root of the ratio between the minor and major axis of the PTV, with values ranging between 0 (maximal elongation) and 1 (sphere-like shape) [22]. As increasing values are associated to poorer survival, this means that spherical PTVs seem less favorable in terms of survival compared to elongated PTVs. It should be stressed here that Elongation values in our dataset were relatively high (above 0.57), meaning that the minor axis of the PTV is at least 33% of the major axis. Specific further studies are therefore required to check performance in case of elongated PTVs, i.e. corresponding to small Elongation values.

Higher order features were not linked to significant survival prediction in TBR-based VOIs. Conversely, increasing values of GLCM Joint Entropy in the PTV, which encodes variability in intensity, were shown to decrease survival. The existence of multiple significant features calls for survival prediction models that can better handle multiple radiomics signatures simultaneously, such as the implemented random forest algorithms.

The generalization capabilities were analyzed by investigating the reproducibility of radiomics-based predictions in two testing scenarios, where images reconstructed with the OSEM-3D algorithm were also included. In independent testing, significantly better results were obtained when considering the PTV as VOI (Table 2). In this setting, feature sets that included shape and first order ^18^F-FET PET features obtained more consistent results, thus confirming the findings of univariate Cox models. The Cox regression models resulted in general lower performance than the implemented Random Forest predictors. When testing the univariate Cox models on OSEM-3D data, the C-index for the Major Axis Length in VOI16 and VOI14 reached 0.58 and 0.56, respectively.

Conversely, the performance in CV when mixing different images (OSEM-2D vs. OSEM-3D) were inferior, with the exception of the *PC(SH-FO)* dataset for survival classification in the PTV (0.7 AUC). This can be partly explained by the lower degree of reproducibility between VOIs segmented in the OSEM-2D reconstructed images versus the OSEM-3D ones. Results from independent testing on OSEM-3D images indicated that features selected on OSEM-2D images can adequately predict survival on different images. Overall best testing performance was found for the *SH-FO* dataset in classification for the PTV, thus confirming the importance of shape and first order features.

The optimal performance in the PTV shows that also areas of the ^18^F-FET PET image at lower image intensities, i.e. outside of the high intensity region at 1.4–1.6 TBR, may play a key role in radiomics-based survival prediction. This also strengthens the relevance of expert-based contouring for PTV delineation, which can potentially highlight relevant areas of low/medium biological activity, due to the intrinsic relevance in survival prediction.

A comparative analysis of our results with other studies is challenging, due to the lack of extensive literature on the topic. ^18^F-FET PET images have been mostly used for diagnostic purposes, and/or to differentiate progression and treatment-related changes [5, 9]. Carles et al. [8] have reported an analysis of relevant ^18^F-FET PET features in recurrent glioblastoma patients, including also overall survival as clinical endpoint. More complex features were highlighted as significant for recurrent glioblastoma patients, whereas our results suggest that shape and first order features are more predictive for overall survival in primary glioblastoma. Conversely, best results in our study were achieved for the PTV, where the GLCM Joint Entropy turned out to be significant in univariate analysis. This confirms that higher order features are indeed relevant for primary glioblastoma patients, especially when considered within the PTV region.

MR images have been used more frequently to predict survival in a radiomics-based approach. The performance reported in our study based on ^18^F-FET PET images are in line with previously reported outcomes on MRI-based radiomics in glioblastoma [33–38]. These studies include also more recent extensions from traditional radiomics to deep learning models, designed to include traditional risk factors as potential predictors [35]. Therefore, ^18^F-FET PET images are suggested as valuable candidates for the prediction of overall survival in primary glioblastoma patients. The complimentary use of MRI and ^18^F-FET PET images has been recently shown in recurrent glioblastoma patients to derive independent biomarkers of response to treatment [38].

The main limitation in our study is the reduced number of patients, also in reason of the unique characteristics of the analyzed patient cohort, which consists solely of glioblastoma patients receiving ^18^F-FET PET prior to primary radio(chemo)therapy. This motivated the use of stringent criteria for feature selection, but hindered a more extensive analysis of the relevance of single features in survival prediction. For this reason, the univariate analysis was restricted to the *Top* features dataset, which might have underestimated the relevance of complex textural features, as these latter were under-represented in the PCA feature selection procedure (Fig. 2). Reported results indeed confirm the appropriateness of such a selection, as PCA including only SH-FO features outperformed PCA including more complex features (Table 2). As further limitation, no comparison with MRI-based radiomic features has been carried out in this work. Different studies have investigated the use of quantitative features from MRI and ^18^F-FET PET for treatment response and survival [6, 10, 38], highlighting the complimentary role of these imaging modalities. A comparative radiomics-based analysis is therefore suggested to highlight the need of dedicated imaging protocols for glioblastoma survival prediction.

## Conclusions

The findings of feature analysis and prediction performance based on ^18^F-FET PET images show that shape features in *voi14*, *voi16,* and PTV are significant predictors of overall survival in glioblastoma patients. When radiomics-based prediction is enlarged to the PTV a more robust prediction of survival is achieved, with 76% accuracy in independent testing both for classification and regression analyses. These preliminary results should be extended to a larger patient cohort for further validation of our findings.

## Data Availability

Due to privacy and ethical concerns, the source data cannot be made available.

## Abbreviations

AUC: Area under the curve
CT: Computerized tomography
FET: Fluorethyltyrosin
FLAIR: Fluid attenuated inversion recovery
FO: First order
GLCM: Gray level co-occurrence matrix
GLDM: Gray level dependence matrix
GLRLM: Gray level run length matrix
GLSZM: Gray level size zone matrix
IDH: Isocitrate DeHydrogenase
MAE: Mean absolute error
MRI: Magnetic resonance imaging
MSE: Mean square error
NGTDM: Neighbouring gray tone difference matrix
OSEM: Ordered subset expectation maximization
PC: Principal component
PCA: Principal component analysis
PET: Positron emission tomography
PTV: Planning target volume
SD: Standard deviation
SH: Shape
SUV: Standardized uptake value
TBR: Tumor to BackgRound
TMZ: TeMoZolomide
VOI: Volume of interest
